# Enhanced stent visualization system for percutaneous coronary intervention in patients with chronic kidney disease: effects on contrast media volume and radiation exposure

**DOI:** 10.1186/s12872-022-02930-0

**Published:** 2022-11-16

**Authors:** Yunpeng Tian, Xiangdong Zhao, Yang Yang, Xiaoshu Cai, Lian Jian, Suzhen Guo, Dasheng Xia, Xin Chen, Chao Li, Qianyu Guo, Bingwei Chen, Chengzhi Lu

**Affiliations:** 1 Department of Cardiology, Tianjin First Central Hospital, Tianjin, China; 2 Advanced Therapies, Siemens Healthineers Ltd., Shanghai, China

**Keywords:** Enhanced stent visualization system, Percutaneous coronary intervention, Chronic kidney disease, Contrast media, Radiation

## Abstract

**Objective:**

We aimed to assess the impact of using enhanced stent visualization (ESV) systems on contrast media volume and radiation dose in percutaneous coronary intervention (PCI), especially for patients with chronic kidney disease (CKD).

**Background:**

Coronary heart disease (CHD) is associated with chronic kidney disease (CKD), as they share a similar pathological pathway. In addition, the iodinated contrast media used for angiography is a risk factor for contrast-associated acute kidney injury (CA-AKI), which could aggravate the progression of CKD. We hypothesized that ESV systems have the potential to reduce the use of contrast media as well as the radiation dose; however, few studies have reported the impact on contrast media with the use of ESV systems.

**Methods:**

We retrospectively collected 124 patients with acute coronary syndrome who underwent PCI from May 2020 to July 2021. The patients were divided into the ESV-guided group (*n* = 64) and angiography-guided group (*n* = 60). Procedural parameters, including contrast media volume, radiation exposure (in Air Kerma-AK and Dose Area Product-DAP), number of cines, cine frames, fluoroscopy and procedure time, were recorded and analysed.

**Results:**

The groups were comparable regarding the patient characteristics. There was a significant reduction in contrast media volume (174.7 ± 29.6 ml vs.132.6 ± 22.3 ml, *p* = 0.0001), radiation exposure (776 (499 - 1200) mGy vs. 1065 (791 - 1603) mGy, *p* = 0.002 in AK; 43 (37 - 73) Gycm^2^ vs. 80 (64 - 133) Gycm^2^, *p* = 0.030 in DAP) and procedure time (53.06 ± 21.20 min vs. 72.00 ± 30.55 min, *p* = 0.01) with the use of ESV systems. Similar results were observed in the subgroup analysis for the patients with CKD.

**Conclusion:**

This study suggested that the use of ESV is associated with reduced contrast media usage, radiation dose and procedure time during PCI. The same results were observed in a subgroup analysis in patients with CKD, and this shows that ESV-guided PCI has the potential to reduce renal impairment and mitigate the progression of CKD for those CHD patients with CKD.

## Introduction

With the rapid development of urbanization, dramatic lifestyle changes and the accelerated ageing of the population, the prevalence of coronary heart disease (CHD) in China has been continuously increasing annually, rising from 4.6‰ in 2003 to 10.2‰ in 2013 [1]. It is estimated that approximately 11 million patients suffer from CHD in China, according to an epidemic report of cardiovascular diseases in 2018 [2]. To improve the survival rate and quality of life for these patients, percutaneous coronary intervention (PCI) has been widely used in the treatment of CHD as a primary method of revascularization[3]. Although patients benefit tremendously from this interventional treatment, iodinated contrast media, inevitably used for X-ray-based imaging, is typically associated with a risk of contrast-associated acute kidney injury (CA-AKI)[4]. Additionally, hypertension, diabetes, dyslipidaemia, and other risk factors for CHD are also common contributing factors for chronic kidney disease (CKD), as both CHD and CKD share similar pathological pathways, such as arteriosclerosis[5]. CA-AKI, a risk modifier leading to the progression of CKD, may cause impaired outcomes in CHD and an increased risk of prolonged hospitalization and early mortality[6, 7]. On the other hand, radiation exposure has been a concerning problem for invasive cardiac procedures in the last decade[8, 9]. Reports have shown that interventional cardiac procedures may lead to a high radiation dose for patients and physicians, which potentially causes deterministic effects on their skin[10, 11]. Therefore, reducing the need for contrast media and radiation exposure while ensuring surgical safety and image quality is critical during PCI. Recently, an enhanced stent visualization (ESV) system (CLEARstent and CLEARstent Live; Siemens Healthineers, Germany) was developed and deployed, providing a clear display of stent deployment by fade in/fade out imaging (CLEARstent) and real-time stent enhancement (CLEARstent Live). Several studies have demonstrated the feasibility, utility and efficacy of this system in facilitating PCI procedures, and this system has been associated with better clinical outcomes[12–16]. However, there are limited studies on contrast media usage with the use of the ESV technique during PCI for patients with CKD complications. In this study, we aimed to evaluate the impact of the ESV system on contrast media usage and radiation dose in PCI procedures, especially for CHD patients with CKD.

## Material and methods

### Study population

The present comparative, retrospective study collected a total of 124 patients with acute coronary syndrome (ACS) who underwent PCI in the Department of Cardiology of Tianjin First Central Hospital from May 2020 to July 2021. One interventional cardiologist with over two years of experience in ESV systems performed stent implantation for all these patients. Patients were eligible for enrolment if they met the following criteria: [1] age < 80; [2] coronary angiography confirmed at least significant stenosis (> 75% stenosis) requiring PCI with stent implantation; [3] diagnosis of unstable angina, ST elevate acute coronary syndromes (STEACS) and non-ST elevate acute coronary syndromes (NSTEACS); and [4] ability to recognize and cooperate, willing to participate in postoperative follow-up, and sign informed consent voluntarily.

Patients were excluded if they [1] had left main or multibranch lesions with valvular disease or ventricular aneurysm requiring bypass surgery rather than interventional PCI; [2] were referred for PCI without stenting or noncoronary interventions, which is beyond the indications of this study; [3] had diseases of the blood system, coagulation disorders or neutropenia, as they were not able to take antiplatelet drugs after stent implantation; [4] had chronic total occlusion treatment, in which the procedure time and contrast media use varies widely with lesion severity, increasing the heterogeneity of the study; [5] underwent intravascular ultrasound and/or pressure wire during the procedure, which may affect homogeneity of the study; [6] underwent PCI with haemodynamic instability requiring implantable temporary pacemaker and/or intra-aortic balloon pump, which may lead to additional radiation doses beyond PCI; [7] were not willing to sign informed consent; [8] had any complications during PCI (e.g., no-reflow, slow-reflow, or dissection), that may result in additional radiation dose and use of contrast media, with high individual heterogeneity; or [9] had a contrast agent allergy, who were not suitable for PCI. Patients were withdrawn if they [1] failed to complete the study and follow-ups; [2] participated in other clinical studies, which may affect the results of this study; and [3] had incomplete patient data, affecting the efficacy or safety evaluation. This study was approved by the institutional review board of the institution in which the procedures were performed.

### Procedures

All PCIs were performed under the radial arterial approach in accordance with the normal routine protocol using an Artis Q.zen X-ray system (Siemens Healthineers, Germany), which is equipped with ESV systems (CLEARstent and CLEARstent Live, Siemens Healthineers). The patients received coronary angiography (CAG) through the right radial access using the standard Judkins method with 4–5 ml iso-osmolar contrast agent to check the left and right coronary arteries. The CAG results were evaluated by two independent cardiovascular radiologists. The operator determined the size and type of the guidewire, wire and predilation balloon according to these CAG results. After injecting nitroglycerin, the target vessel and lesion were evaluated by angiography. The operator selected the size of the stent based on the diameter of the vessel. Normally, postdilation would be performed after the stent was deployed correctly. All patients regularly took aspirin 100 mg/d, clopidogrel 75 mg/d (or ticagrelor 180 mg/d), statins, β-blockers, ACEIs, nitrates, etc., after the procedure.

### ESV system-guided stent implantation

The ESV system was processed based on a balloon catheter with radiopaque markers in the region of interest. According to these makers, automatic registration will be performed of all frames within the acquired sequence. During the procedure, fluoroscopy or cine images exceeding 25 frames could be both used for postprocessing by ESV systems for better visualization of the deployed stents. In addition, it allowed real-time visualization of stent placement with 45–60 frames of cine images at a rate of 30 frames/s. The ESV images were displayed on the screen immediately without additional interactions. Typically, no contrast media was used when obtaining the ESV images. An additional 2-3 s of contrast injection would be applied only if the operator needs a higher quality image to capture more details. The use of ESV systems was determined by a certain operator. An ESV system were considered for use under the following circumstances: [1] for accurate positioning of the stent; [2] to determine if the stent is fully expanded; [3] to evaluate if the stent connection is reasonable; [4] to determine the site of wire re-crossing in bifurcation lesions.

### Data collection

All patients received interventional treatment based on coronary angiography and were divided into two groups: an ESV-guided group (*n* = 64) and an angiography-guided group (*n* = 60). Patients in the ESV-guided group used ESV system to evaluate stent positioning, connection and expansion during the intervention. In the angiography-guided group, stent implantation and expansion were evaluated using conventional X-ray images. As a subgroup analysis, the patients with serum creatinine greater than 104 μmol/L in the two groups were selected for CKD analysis.

The patient characteristics were collected and included age, sex, body mass index (BMI), location of the target vessel, and other detailed characteristics related to the complexity of the procedure. Procedure-related data were also collected: radiation exposure, number of cines, cine frames, fluoroscopy and procedure time, as well as contrast media volume. Radiation exposure was recorded in the air kerma (AK) and the dose area product (DAP) during fluoroscopy and in total (in combination of fluoroscopic and angiographic imaging doses). In addition, the number of cines, cine frames, fluoroscopy time and procedure time were collected from the dose report. The procedure time was recorded from the point of the patient on the table.

### Statistical analysis

The patient characteristics and all of the intraoperative parameters were analysed and compared between the two groups accordingly. SPSS 22.0 statistical software was used for data analysis. Categorical data were summarized as frequencies and percentiles using the chi-square test or Fisher's exact probability method. For continuous variables, data are presented as the mean ± standard deviation (SD) for variables normally distributed and as median with interquartile range (IQR) for skewed variables. Normally distributed continuous variables were compared using a 2-sided Student’s t test, and the Mann‒Whitney U test was used for nonparametric variables. Statistical significance was set to 5%.

## Results

### Patient characteristics

During the study period, a total of 124 patients who underwent PCI were included. Of these 124 patients, 64 (51.6%) patients received ESV-guided PCI, and 60 (48.4%) patients received standard PCI under angiography guidance. The mean age of the ESV-guided group was 61 ± 11 years, and 62% were male. Furthermore, the mean age of the angiography group was 62 ± 10 years, and 70% were male. The patient characteristics were comparable between the two groups in age, sex, BMI, lesion types and other vascular parameters (see Table 1).

**Table 1:** Comparison of the patient characteristics between the two groups

Characteristics	ESV-guided Group (*n* = 64)	Angiography-guided group (*n* = 60)	*p* value
Age	61 ± 11	62 ± 10	0.242
Male	37 (58%)	42 (70%)	0.158
BMI	26.2 ± 2.9	25.8 ± 2.7	0.387
No. of lesions treated every PCI	1.2 ± 0.4	1.1 ± 0.3	0.206
*Location of target vessel*			0.753
LM	1 (1%)	0	
LAD	31 (46%)	30 (49%)	
LCX	17 (25%)	13 (21%)	
RCA	19 (28%)	18 (30%)	
*B2/C lesions*			0.760
B2	26 (41%)	26 (43%)	
C	38 (59%)	34 (57%)	
Multivessel PCI	35 (55%)	42 (70%)	0.079
Ostial PCI	2 (3%)	4 (6%)	0.358
Bifurcation PCI	5 (8%)	2 (3%)	0.280
Post-dilatation	55 (86%)	51 (85%)	0.439
No. of stents per PCI	1.3 ± 0.5	1.4 ± 0.6	0.882
Overlapping stents	17 (27%)	18 (30%)	0.671

Values are expressed as median with IQR or number (%)

*ESV* Enhanced stent visualization, *BMI* Body mass index, *PCI* Percutaneous coronary intervention, *LM* Left main arterial, *LAD* Left anterior descending arterial, *LCX* Left circumflex arterial, *RCA* Right coronary arterial

### Procedural characteristics

A statistically significant reduction was demonstrated on contrast media analysis in the ESV-guided group compared to the angiography-guided group (132.6 ± 22.3 ml vs. 174.7 ± 29.6 ml, *p* = 0.0001). With the use of ESV systems, the radiation exposure (in AK and DAP) was significantly decreased for fluoroscopy alone (562 (332 – 919) mGy vs. 840 (628 – 1346) mGy, *p* = 0.002 in AK; 31 (25 - 61) Gycm^2^ vs. 65 (54 - 111) Gycm^2^, *p* = 0.023 in DAP) and for the total amount of fluoroscopic and angiographic imaging exposure (776 (499 - 1200) mGy vs. 1065 (791 - 1603) mGy, *p* = 0.002 in AK; 43 (37 - 73) Gycm^2^ vs. 80 (64 - 133) Gycm^2^, *p* = 0.030 in DAP). In addition, a significantly shorter procedure time was observed in the ESV-guided group, with a 30% decrease (53.06 ± 21.20 min vs. 72.00 ± 30.55 min, *p* = 0.01). No significant difference was shown in the fluoroscopy time, number of cine runs or cine frames (see Table 2).

**Table 2:** Comparison of the procedural characteristics between the two groups

Characteristics	ESV-guided group	Angiography-guided group	*p* value
Air Kerma – fluoro (mGy)	562 (332 – 919)	840 (628 – 1346)	0.002 *
Air Kerma – total (mGy)	776 (499—1200)	1065 (791—1603)	0.002 *
DAP – fluoro (Gycm^2^)	31 (25—61)	65 (54—111)	0.023 *
DAP – total (Gycm^2^)	43 (37—73)	80 (64—133)	0.030 *
Fluoroscopy time (min)	13.00 ± 8.14	14.54 ± 7.30	0.289
Procedure time (min)	53.06 ± 21.20	72.00 ± 30.55	0.01 *
No. of cine runs (n)	22 ± 6	23 ± 8	0.185
Cine frames(n)	821.36 ± 299.93	821.88 ± 294.90	0.924
Contrast media (ml)	132.6 ± 22.3	174.7 ± 29.6	0.0001 *

Values are expressed as median with IQR or mean ± SD

*DAP* Dose area product

### Subgroup analysis for patients with CKD

For the subgroup analysis, similar results as the overall analysis were observed. The patient data were also comparable between the two groups. In the ESV-guided group, the amount of contrast media was significantly reduced by 36% (179.41 ± 16.76 ml vs.114.5 ± 13.56 ml, *p* = 0.0001). Except for a significant half reduction in the radiation exposure in fluoroscopy alone (367 (285 - 664) mGy vs. 864 (553 - 1693) mGy, *p* = 0.001 in AK; 27 (16 - 48) Gycm^2^ vs. 67 (50 - 139) Gycm^2^, *p* = 0.010 in DAP) and the total amount of fluoroscopic and angiographic imaging (615 (415 - 855) mGy vs. 1063 (856 - 2171) mGy, *p* = 0.001 in AK; 39 (35 - 72) Gycm^2^ vs. 84 (70 - 145) Gycm^2^, *p* = 0.013 in DAP), the number of cine runs was also decreased significantly in the ESV-guided group. Additionally, the angiographic-guided group showed a longer procedure time than the ESV-guided group (47.75 ± 15.43 min vs. 65.88 ± 23.81 min, *p* = 0.029). No significant difference was observed in the fluoroscopy time or cine frames (see Tables 3, 4).

**Table 3:** Comparison of the patient characteristics between the two groups of patients with CKD

Characteristics	ESV-guided Group (*n* = 20)	Angiography-guided group (*n* = 17)	*p* value
Age	58 ± 8	61 ± 11	0.243
Male	13 (66%)	14 (82%)	0.236
BMI	26.1 ± 3.4	26.1 ± 1.6	0.721
No. of lesions treated every PCI	1.1 ± 0.2	1.1 ± 0.3	0.157
*Location of target vessel*			0.063
LM	0	0	
LAD	10 (50%)	9 (53%)	
LCX	5 (25%)	0 (21%)	
RCA	5 (25%)	8 (47%)	
*B2/C lesions*			0.402
B2	11 (55%)	7 (41%)	
C	9 (45%)	10 (58%)	
Multivessel PCI	11 (55%)	12 (71%)	0.330
Ostial PCI	0	1 (6%)	0.272
Bifurcation PCI	2 (10%)	1 (6%)	0.674
Post-dilatation	17 (85%)	13 (76%)	0.509
No. of stents per PCI	1.1 ± 0.3	1.5 ± 0.8	0.083
Overlapping stents	3 (15%)	7 (41%)	0.074

Values are expressed as median with IQR or number (%)

*ESV* Enhanced stent visualization, *BMI* Body mass index, *PCI* Percutaneous coronary intervention, *LM* Left main arterial, *LAD* Left anterior descending arterial, *LCX* Left circumflex arterial, *RCA* Right coronary arterial

**Table 4:** Comparison of the procedural characteristics between the two groups in the subgroup analysis

Characteristics	ESV-guided group	Angiography-guided group	*p* value
Air Kerma-fluoro (mGy)	367 (285—664)	864 (553—1693)	0.001 *
Air Kerma-total (mGy)	615 (415—855)	1063 (856—2171)	0.001 *
DAP – fluoro (Gycm^2^)	27 (16—48)	67 (50—139)	0.010 *
DAP – total (Gycm^2^)	39 (35—72)	84 (70—145)	0.013 *
Fluoroscopy time (min)	9.95 ± 5.83	13.52 ± 7.64	0.309
Procedure time (min)	47.75 ± 15.43	65.88 ± 23.81	0.029 *
No. of cine runs (n)	19 ± 4	25 ± 9	0.026 *
Cine frames(n)	686.40 ± 158.51	857.94 ± 357.89	0.102
Contrast media (ml)	114.5 ± 13.56	179.41 ± 16.76	0.0001 *

Values are expressed as median with IQR or mean ± SD

*DAP* Dose area product

## Discussion

The present study retrospectively evaluated the impact of ESV systems on the contrast media volume and radiation exposure for patients who underwent PCI procedures. To the best of our knowledge, we are the first to report the comparative results of contrast media usage between the group guided by an ESV system and the group guided by standard angiography. A significant reduction in the contrast media volume, radiation exposure (in AK and DAP) and procedure time was shown with the use of ESV systems. Similar results were observed in the subgroup analysis for the patients with CKD.

The ESV system is a fluoroscopic-based technique that can provide enhanced visualization of stents with adequate imaging for diagnosis and guidance. Studies have shown that it increases the amount and quality of information during PCI to facilitate the identification of bifurcation stenting, precise stent positioning, stent underexpansion and defining stent-vessel wall relationships in case reports[12, 13, 17, 18]. Biscaglia et al.[15] demonstrated a novel timing of intraoperative stent fracture identification during the index PCI with the systematic application of ESV. In addition, the ESV system was suggested to be effective in reducing excessive overlap in the implantation of bioresorbable vascular scaffolds[14]. Furthermore, the results in the study of McBeath et al. [16] showed significantly improved correlations of an ESV system and the clinical outcome. Jin et al. [19] pointed out that the use of an ESV system (Stentboost, Philips) can be performed with a comparable radiation dose as conventional X-ray fluoroscopy imaging and suggested that the operator’s experience has an important impact on the radiation dose. However, there is a paucity of research that examines contrast media usage during PCI using such a technique.

The current reliable way to perform PCI with the minimum amount of contrast or even without contrast administration is by using the guidance of intravascular ultrasound imaging (IVUS), which helps to identify the lesion and its length[20]. Nevertheless, due to its time-consuming nature, expense and requirement for specifically trained personnel, this technique is not routinely used in daily practice [21]. In contrast, as an alternative option, ESV systems such as the CLEARstent technique only require a few seconds of fluoroscopy or cine images and immediately provide real-time visualization of the stent. With enhanced visualization, stent positioning, deployment and overlapping could be performed in a clear and intuitive way without the need for repeated contrast, thereby greatly lowering the use of contrast media. Especially for complex cases, such as bifurcation lesions, the relationship between the stent and wire and the detection of the recrossing wire could be evaluated and facilitated with only one fluoroscopy image, omitting repeated enhanced acquisitions to make confirmation. Furthermore, underexpansions could also be detected without contrast injection, which helps physicians perform proper postdilation in a minimum amount of contrast medium. As a result, in this study, the use of ESV systems demonstrated a significant reduction in contrast media usage compared with the traditional angiography-guided procedure for preventing the occurrence of contrast-induced nephropathy. In the subgroup analysis, for patients with existing CKD, a similar result on contrast media suggested that ESV-guided PCI has the potential to reduce renal damage and mitigate the progression of CKD, which is of great significance for these patients.

Unlike the result of a nonsignificant impact on patient radiation dose in the study of Jin et al.[19], who also noted a learning curve of Stentboost imaging and radiation protection, our study found a significant reduction in the radiation dose and procedure time in the ESV-guided group, while maintaining high-quality visualization with an optimization of stents. In traditional angiographic procedures, several cines are required to obtain adequate stent visualization, while fewer cine shots are needed for ESV-guided procedures, as enhanced visualization of stent deployment eliminates unnecessary radiation exposure. In addition, since fluoroscopy images could also be postprocessed using the ESV system developed by Siemens Healthineers, the radiation dose could be greatly reduced in terms of replacing the regular cines with fluoroscopy. Not only could the reduced radiation dose provide relative radiation protection for patients and operators, but the shortened procedure time could also reduce the development of heart failure from prolonged surgical trauma, especially for patients with CKD.

Regarding to the occurrence of AKI, a meta-analysis [22] investigating nine studies with 32 181 patients showed a significantly lower incidence of AKI with radial access compared to femoral access in patients underwent PCI. One of the possible explanations of nephron-protective effects of radial access might be mediated by the reduced rate of major bleeding and access-site complications [23]. Gragnano et al. [24] compared the clinical outcomes between radial crossover and femoral crossover, and the results lend support to use of the radial access as the default approach in patients with ACS. In our center, transradial access was routinely performed as the preferred vascular access site in patients underwent PCI, and all the patients in this study underwent successful radial access in both groups. Currently, strategies to avoid AKI in CHD patients with existing CKD is limited [25]. The combination uses of radial access and ESV systems guidance could be an alternative tool by prevention of renal hypoperfusion with radial access and contrast volume minimization.

**Fig. 1:**
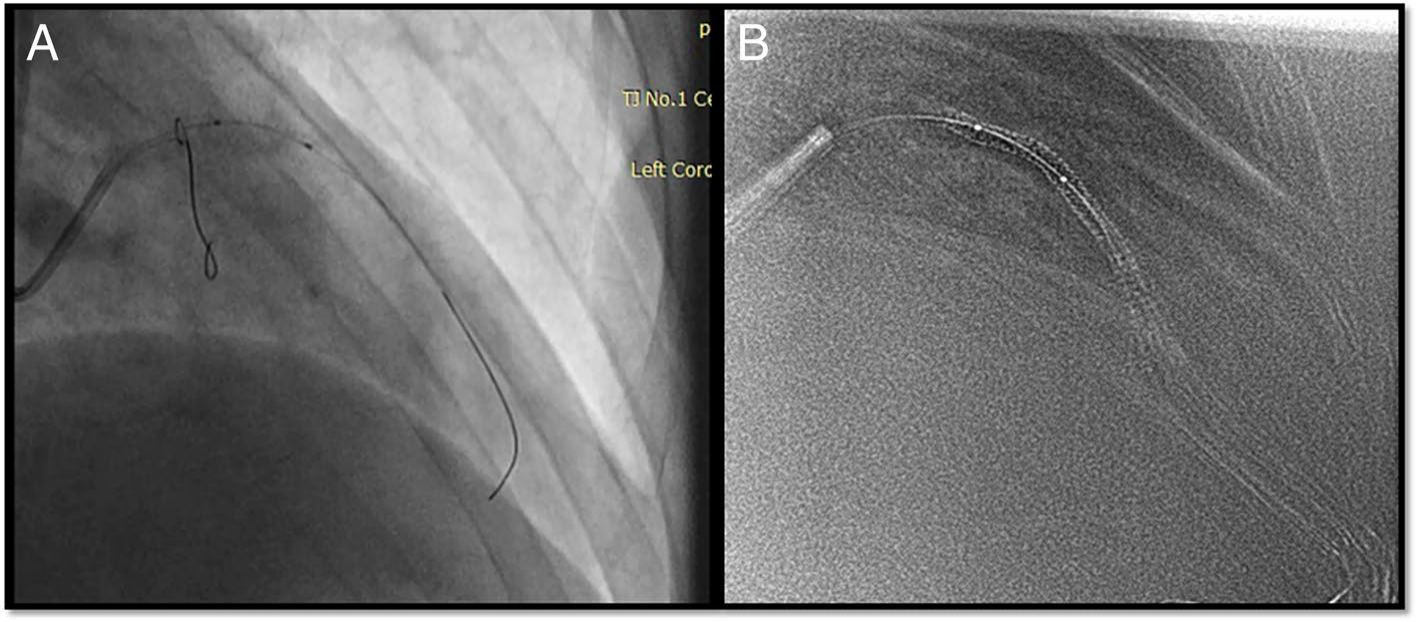
The ESV system is used to visualize the drug-eluting stent. **A** Drug-eluting stent deployment under conventional fluoroscopy; **B** Clear visualization under the guidance of the ESV system

**Fig. 2:**
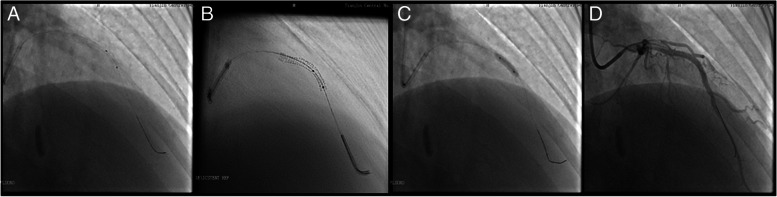
The ESV system is used to detect stent underexpansion and guide postdilation. **A** Stent deployment under conventional fluoroscopy; **B** The ESV system helped detect underexpansion of the stent and assisted in aligning the balloon marker with the stent margin; **C** Postdilation under fluoroscopy; **D** Angiography confirmation after deployment

**Fig. 3:**
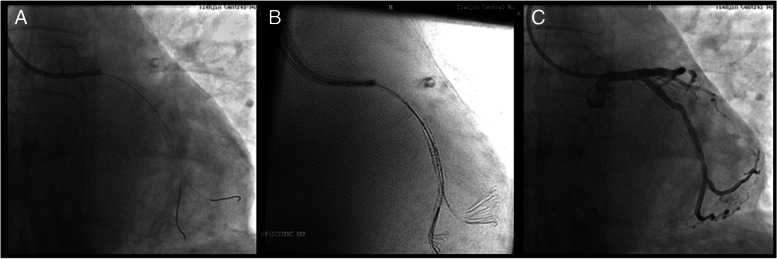
The ESV system is used to guide stent deployment for bifurcation lesions. **A** Stent deployment under conventional fluoroscopy; (**B)** Stent deployment under the guidance of the ESV system to help ensure that the wire crossed the distal cell; (**C**) Angiography confirmation after deployment

**Fig. 4:**
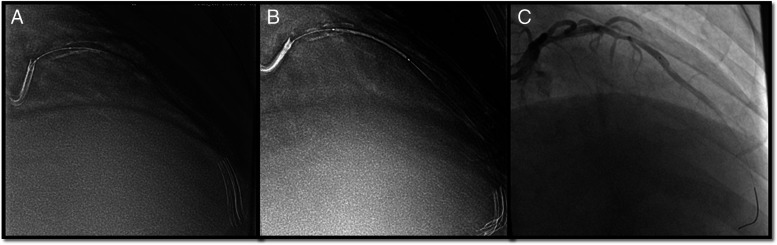
The ESV system is used to identify vascular calcification. **A** Identification of calcification to guide predilation under ESV images; (**B**) The ESV system assists stent positioning with visualization of severe calcification; (**C**) Angiography without enhanced visualization when calcification is barely distinguished

Studies [19, 26] pointed out that sufficient experience of the surgeon in using a similar technique has an important impact; however, we found a steep learning curve for the Siemens ESV technique. On the other hand, despite an inevitable learning curve of ESV systems, it could shorten the overall learning curve of percutaneous coronary intervention procedures, especially for junior operators. Additionally, it offers some obvious advantages, which are as follows. [1] ESV systems improved the visualization of drug-eluting stents. In recent years, the appearance of some new drug-eluting stents has made it more difficult to locate and deploy them by traditional angiography methods due to their low relative thickness and X-ray impenetrable materials. ESV systems could significantly improve the positioning and imaging of this self-dilating stent (see Fig. 1). [2] ESV systems enhanced the identification of stent underexpansion (see Fig. 2). Incomplete or uneven expansion of stents is the main risk factor affecting short-term and long-term efficacy, as is the case in the era of drug-eluting stents. Timely identification of incomplete expansion of stents is a prerequisite to avoid risks. At present, IVUS is the gold standard for the evaluation of stent structure and dilation level. Under the angiographic-based method, contrast media is required to acquire adequate evaluation of stents. As an additional option, ESV systems require no additional contrast media or hardware and provide instant information about its structure. [3] The ESV system provided real-time guidance for bifurcation lesions (see Fig. 3). For the treatment of bifurcation lesions, the use of distal cells when rewiring was recommended by The European Club to improve procedural outcomes. Angiography and IVUS are unreliable at detecting the site of wire recrossing. Optical coherence tomography (OCT) has its own advantage in assessing rewiring; however, additional contrast is also needed. ESV systems can enhance the signal of stent struts and increase the visibility of stents to estimate the wire location, with no additional contrast needed. [4] ESV systems increased the identification of vascular calcification. Coronary calcification hinders the placement of stents because of potential inadequate stent expansion and difficult transition. However, it is usually difficult to distinguish the stent struts and calcification under standard angiography. ESV systems could identify the distribution of calcification with or without a stent being present and distinguish stents from wall calcification. As a result, the reason for insufficient stent expansion could be confirmed immediately to prevent vascular rupture in cases of overexpansion (see Fig. 4). [5] Intracoronary imaging techniques, such as IVUS or OCT, have a fundamentally different purpose than angiography. ESV imaging, which was based on angiography, do not provide the same accurate information as IVUS or OCT. However, ESV systems could be cost and time saving, as well as easy-to-use compared to those techniques. By providing an immediately usable improved images, ESV systems could be utilized as an alternatively or complementary tool for interventional cardiologists.

We should acknowledge some limitations. First, this is a single-centre retrospective study with a small sample size; thus, a larger, multicentre prospective study will be needed in the future. Second, automatic marker detection may misidentify other objects as markers, and an additional step of magnification may be needed to improve visualization. Third, there is a need to further address clinical outcomes after the use of ESV systems with a longer follow-up, especially for patients with CKD. Furthermore, in the absence of the incidence of AKI in this study, any conclusion on the risk reduction of AKI due to ESV system-guided PCI is speculative. Adequately powered studies showing the association between reduction in the risk of AKI and use of ESV systems are needed.

## Conclusions

This study suggested that the use of ESV is associated with reduced contrast media usage, radiation dose and procedure time during PCI. The same results were observed in the subgroup analysis in patients with CKD, indicating that ESV-guided PCI has the potential to reduce renal impairment and mitigate the progression of CKD for those CHD patients with existing CKD.

## Data Availability

The datasets used and/or analysed during the current study are available from the corresponding author on reasonable request.

## Abbreviations

ESV: Enhanced stent visualization
PCI: Percutaneous coronary intervention
CKD: Chronic kidney disease
CHD: Coronary heart disease
CA-AKI: Contrast-associated acute kidney injury
ACS: Acute coronary syndrome
STEACS: ST elevate acute coronary syndromes
NSTEACS: Non-ST elevate acute coronary syndromes
CAG: Coronary angiography
BMI: Body mass index
AK: Air kerma
DAP: Dose area product
